# On the prospects of basal cognition research becoming fully evolutionary: promising avenues and cautionary notes

**DOI:** 10.1007/s40656-025-00660-y

**Published:** 2025-02-06

**Authors:** Alejandro Fábregas-Tejeda, Matthew Sims

**Affiliations:** 1 Centre for Logic and Philosophy of Science, Institute of Philosophy, KU Leuven, Leuven, Belgium; 2 Leverhulme Centre for the Future of Intelligence, University of Cambridge, Cambridge, UK; 3 Institute for Philosophy II, Ruhr-University Bochum, Bochum, Germany

**Keywords:** Basal cognition, Development, Evo-Devo, Evolution, Cognitive capacities

## Abstract

The research programme ‘basal cognition’ adopts an evolutionary perspective for studying biological cognition. This entails investigating possible cognitive processes in ‘simple’–often non-neuronal–organisms as a means to discover conserved mechanisms and adaptive capacities underwriting cognition in more complex (neuronal) organisms. However, by pulling in the opposite direction of a tradition that views cognition as something that is unique to neuronal organisms, basal cognition has been met with a fair amount of scepticism by philosophers and scientists. The very idea of approaching cognition by way of investigating the behaviour and underlying mechanisms in, say, bacteria, has been seen as preposterous and harmful to both cognitive science and biology. This paper aims to temper such scepticism to a certain degree by drawing parallels with how the evolution of ‘development,’ another loaded concept that refers to a not-so-easily definable, contested bundle of phenomena, has been fruitfully approached in Evolutionary Developmental Biology (Evo-Devo). Through this comparison, we identify four promising features of the basal cognition approach. These features suggest that sweeping scepticism may be unwarranted. However, each of them comes with important epistemic cautionary notes that should not be disregarded. By presenting these twofold considerations as potential ways to integrate a fully evolutionary perspective into basal cognition, this paper seeks to provide clarity and direction for the advancement of this research programme.

## Introduction: two approaches for addressing the scope of cognition

In the philosophy of cognitive science and cognitive science more generally the following fundamental scope question arises: “where does biological cognition begin?”. By addressing which kinds of organisms exhibit cognition and identifying some shared feature common to such organisms this question is thought to reveal something important about the nature of cognition. There have been two general (yet non-exhaustive) ways that this scope question has been approached. The first is a *definition-first approach*. It starts off with the assumption that cognition refers to a general process and then proceeds in introducing a criterion (or criteria) that demarcates cognition from other non-cognitive biological processes such as metabolism, physiological regulatory processes and the like. This demarcation criterion, usually in the form of (a) necessary and/or sufficient condition(s), is then used to adjudicate whether or not some specific kind of organismal behaviour is or is not cognitively driven. Such an approach to answering the scope question is usually taken up by philosophers and framed in terms of identifying “the mark of the cognitive” or, similarly, singling out some necessary feature that satisfies the requirements for “minimal cognition.”

There have been numerous suggestions as to what such marks are, ranging from having representational states with intensionality (Adams, 2018) to exhibiting sensorimotor coordination (van Duijn et al., 2006) or future-oriented intentional dynamics (Sims, 2021). Whether or not philosophical debates about the mark of the cognitive or minimal cognition reveal anything about the nature of biological cognition other than one’s pre-theoretical commitments regarding cognition remains to be seen (Facchin, 2023). To date, this manner of addressing the scope question has proven itself to be reminiscent of the many-headed hydra immortalised in Greek myth; any attempt to answer it once and for all has tended to stubbornly give rise to more questions of similar or same form requiring even more criteria and/or more nuancing of old criteria ad infinitum. That said, definition-first approaches can often serve instrumentally as a means to rouse interest in the general notion of cognition, enlisting more soldiers in the battle to slay the many-headed hydra. Needless to say, the hydra is still alive and slithering.

There is another manner of addressing the scope question, however, which purports to be prima facie more promising. Eschewing the search of the mark of the cognitive, it takes an *evolutionary approach*. Accordingly, a more thorough articulation of the question might be: “how far back can we traverse the phylogenetic tree and identify instances of biological processes that are functionally analogous to and/or in historical continuity with those processes that have been investigated within the domain of traditional cognitive science?”. Central to this approach is an evolutionary assumption; namely, many of the capacities that are found in phyletically younger (more recently evolved) taxa like us have functional predecessors in older taxa from which they have evolved. Some of these older capacities and/or their underlying causal mechanisms, the assumption goes, have been evolutionarily conserved due to their contribution to survival and fecundity (i.e., fitness). With this assumption to hand, evolutionary reasoning-first approaches look to investigate specific instances of putative cognitive capacities in extant organisms that are phylogenetically distant from us (e.g., slime moulds, carnivorous plants, ctenophores, etc.). Working with organisms from early diverging lineages, they aim to identify common principles and conserved cognitive mechanisms that are shared between structurally and functionally simpler organisms and those that are more complex according to certain metrics (e.g., multicellular organisms with a diversity of cell types and cell functions). This latter way of framing the scope question is part of a venerable interdisciplinary tradition, dating at least as far back as to eighteenth- and nineteenth-century evolutionism (Richards, 1987).

In recent years, this approach to investigating cognition has become increasingly more popular (e.g., Barron et al., 2023; Beran et al., 2014; Chittka et al., 2012; Godfrey-Smith, 2020) and has been marshalled in the research programme dubbed “basal cognition” (Levin et al., 2021; Lyon et al., 2021).^1^ For the purposes of getting empirical investigation off the ground, basal cognition researchers conceptualise biological cognition as a toolkit of adaptive capacities that have been shaped and maintained by commonplace evolutionary processes, including Darwinian evolution by natural selection. Formulated so as to be phyletically neutral, the basal toolkit is based upon various capacities that have previously been observed in bacteria (Lyon et al., 2021).^2^ These include (but are not limited to) memory, learning, decision-making, perception, anticipation, valence, and behaviour (Lyon et al., 2021). In recognising that each of these capacities have been shaped by natural selection and other evolutionary forces, and are thus fundamentally biological processes, a particular emphasis is placed on taking an evolutionary perspective on cognition. What sets basal cognition apart from a purely conceptual manner of answering the scope question is its focus on theories that generate (in principle) testable hypotheses (Levin, 2022; Sims, 2024a). In this regard, it prioritises experimental results over a priori reasoning about criteria for cognition, evaluating hypotheses based on empirical findings.

This willingness of biologists, cognitive scientists, and philosophers to deploy a bottom-up, *explananda*-steered approach (see also Waal & Ferrari, 2010) is a significant departure from a long-standing neuro-centric (or more generally zoo-centric) view of cognition, one that often informs or is the basis for the a priori criteria that have sustained the presence of the many-headed hydra. And unlike the mark of the cognitive, definition-first approach to answering the scope question, basal cognition has yielded some promising (yet non-conclusive) results bearing on understanding conserved cognitive capacities and mechanisms (see the 2021 basal cognition double special issue in the *Philosophical Transactions of the Royal Society B*; for an overview, see Levin et al., 2021; Lyon et al., 2021).

Pulling in the opposite direction of a tradition that views cognition as something that is unique to neuronal organisms, the basal cognition programme has been met with a fair amount of scepticism by some philosophers and biologists (Figdor, 2022, 2024b; Loy et al., 2021; Mallatt et al., 2023). For example, it is argued that there is no irrefutable evidence that what is being called “learning” or “memory” in non-neuronal organisms is remotely similar to what cognitive scientists are interested in when investigating learning and memory (Loy et al., 2021). This form of scepticism aims at driving a wedge between bona fide cognitive capacities as uniformly recognised by cognitive science and other processes that are only superficially similar to those capacities that cognitive science focuses upon. Another form of scepticism draws attention to the abstract nature of the models used by some basal cognition researchers. Figdor (2024b) contends that even when scientists are able to instrumentally employ the same type of (mathematical and computational) models for capturing the adaptive flexibility of non-neuronal organisms’ behaviours (e.g., by modelling them as feedback control mechanisms), the phylogenetic and conceptual relationships of these feedback-controlled behaviours to those of organisms we already accept as cognitive is left out of the models. And yet another form of scepticism rests on the very meaning of “cognition.” If cognition is defined as necessarily involving mental states that have a specific kind of representational format (e.g., exhibiting intensionality), then the very idea of approaching cognition by way of investigating the behaviour and underlying mechanisms in, say, bacteria, is seen as preposterous and harmful to both cognitive science and biology (Adams, 2018).

This paper aims to challenge such scepticism to a certain degree by drawing parallels with how the evolution of *development*, another loaded concept that refers to a not-so-easily definable bundle of phenomena, has been approached in the now-consolidated, empirically profitable field of evolutionary developmental biology (Evo-Devo). Development, just as cognition, is one of those umbrella scientific concepts whose univocal characterization has proven elusive and disagreement abounds (Pradeu et al., 2016), not to mention that no overarching “theory of development” appears to be in sight (Minelli & Pradeu, 2014). Although the question of establishing a definition-first approach to development seemed to be pressing during the 1950s when embryology transformed into ‘developmental biology’ (Burian & Thieffry, 2000; see also Hopwood, 2019; Pradeu et al., 2016), definitional concerns eventually receded into the background as empirical research took off, and a comprehensive conceptualization of ‘development’ was not seen as a prerequisite for the emergence of Evo-Devo in the 1980s, which was also highly contentious among evolutionary biologists in its beginnings and well into the early 2000s (Amundson, 2005).

The resemblance between the evolutionary and comparative study of development and cognition lies not only in the seemingly insurmountable difficulties of reaching a satisfactory definition that encompasses the varied phenomena to which their extensions purportedly refer (which could then be projected to solve scope problems),^3^ but also in that many of the same types of questions we can ask about the evolution of development and developmental repertoires can be asked of the evolution of cognition and cognitive capacities. This includes, among others: How many times have they evolved in phylogenetic history? Which cognitive or developmental traits are homologous or homoplastic *in which* taxa? Could certain cognitive or developmental processes in a particular organism be *sui generis* due to its prior evolutionary trajectory or are they rather indicative of a larger lineage or clade?

Through our comparison between how ‘development’ has been approached from an evolutionary perspective in Evo-Devo, we identify four specific parallels with the basal cognition approach that bear upon its prospects for becoming a fully evolutionary research field that is able to address the scope problem: First, both fields conduct comparative causal-mechanistic investigations to uncover shared developmental or cognitive toolkits and capacities (Sect. 2). Second, researchers in each area assume *panextensionalist* positions about the phylogenetic scope of ‘cognition’ and ‘development’ in an attempt to counteract purported evolutionary biases of *oligoextentionalist* positions that only grant cognition or development to few organisms (i.e., neuronal metazoans and clonal multicellular organisms, respectively) (Sect. 3). Third, when investigating developmental and cognitive traits, sound phylogenetic thinking can aid in distinguishing between homologies and homoplasies, as well as in countenancing convergence in evolutionary scenarios (Sect. 4). Fourth, by adopting a fully evolutionary perspective, the loss, gain, and uniqueness of particular developmental and cognitive traits and capacities should be studied in particular lineages (Sect. 5).

It should be said that these parallels are not categorially equivalent for basal cognition as-it-is-presently-practiced: The first two parallels are descriptive of current work in basal cognition research, while the last two have a normative bent regarding the considerations that scientists could bear in mind when adopting a bona fide evolutionary perspective (e.g., in terms of methodology or inference generation and justification). Importantly, however, is that each parallel signals promising features of the basal cognition approach. As such, these features suggest that sweeping scepticism against it may be unwarranted. This notwithstanding, each of them also comes with important cautionary notes, attention to which we believe will ultimately improve basal cognition’s standing as an evolutionarily grounded approach to investigating cognition. By presenting these twofold considerations as potential ways to integrate a fully evolutionary perspective into basal cognition, we seek to provide clarity and direction for the advancement of this research programme. In this sense, and more generally speaking, we intend to show how evolutionary reasoning-driven approaches to tackle the problem of the scope of cognition can be strengthened and pursed further.

## Shared toolkits and causal-mechanistic research

Ever since its inception, one of the goals of Evo-Devo has been to uncover shared genetic-developmental toolkits in phylogenetically distant organisms, and this is now taken to be a rather uncontroversial scientific pursuit. However, in the 1980s, one of the first discoveries of such shared developmental underpinnings took the scientific community by surprise: the finding that *Hox* genes, first characterized as the genes of the *Bithorax* and the *Antennapedia* complexes of *Drosophila melanogaster* which have homeotic effects when mutated (e.g., fruit flies with an extra pair of wings instead of halteres or with ectopic legs instead of antennae), exist across many animal groups (McGinnis et al., 1984a, 1984b). These are transcription factors, later found in other eukaryotic groups as well, which contain a 180-base pair sequence (the homeobox) that encodes a region called the homeodomain. In a large range of bilaterian metazoans, this region binds to DNA to regulate the expression of downstream genes related to anterior–posterior patterning. Part of the surprise instigated by the discovery of the widespread presence of homeobox-containing genes was due to theoretical expectations from evolutionary biologists and the state of knowledge at the time on what was presumed to be the genetic basis of adaptation. For instance, in a representative statement, the renowned evolutionary biologist Ernst Mayr (1963) stressed: “Much that has been learned about gene physiology makes it evident that *the search for homologous genes is quite futile* except in very close relatives” (p. 609; emphasis added).

In alignment with these conceptual presuppositions, searching for shared genes that partake in the morphogenesis of organic structures was not even contemplated as a worthwhile investigative route. However, just as with the case of the *Hox* genes and anterior–posterior patterning, soon other orthologous genes involved in other developmental processes, for instance, in eye and appendage formation (Halder et al., 1995; Shubin et al., 1997), were found across invertebrate and vertebrate species in the wake of what we now call modern ‘Evo-Devo’ research. This relatively small set of key regulatory genes (e.g., transcription factors and components of paracrine signalling pathways) related to conserved developmental patterning functions for tissues, organs, and body axes throughout the diverse phyla of bilaterally symmetrical animals came to be known as “the genetic tool-kit” (Wilkins, 2013). Along these lines, the “ancestral complexity” of genomic repertoires came to be hailed as one of the key theoretical tenets of Evo-Devo (Carroll, 2008).

The quest for “master control genes” (Gehring, 1998), which proved to be important for the expansion of concepts such as “deep homology” (Shubin et al., 1997, 2009) and the phylogenetic screening of shared developmental genes, soon was found to be overly simplistic and gave way to a more nuanced approach. This shift involved the characterization and modelling of gene regulatory networks involved in character formation (Davidson, 2006; for discussion, see Morange, 2014) and, in general, more integrative appraisals of the causal-mechanistic basis of development. From its proximal origins to the present day, Evo-Devo has been in the business of providing causal-mechanistic explanations of development and its evolution. Beside gene regulatory networks underlying specific character formation, Evo-Devoists investigate different forms of ‘developmental repatterning’ (Arthur, 2010), generic mesoscale physical forces and dynamical patterning modules (Newman & Bhat, 2009), epigenetic mechanisms (Jablonka & Lamb, 2007), cell and tissue interactions, and other phenotype-generating mechanisms at different levels of biological organization (Salazar-Ciudad & Jernvall, 2013).

Like Evo-Devo’s search for developmental mechanisms, basal cognition research is currently in the business of searching for causal-mechanistic realisers of the various capacities comprising the basal cognitive toolkit. In basal cognition, scientists are investigating intracellular and intercellular mechanisms such as signal transduction (Lyon, 2015), cell–cell signalling (Yang et al., 2020), oscillations (Boussard et al., 2021; Hanson, 2021), cell networks and circuits (Rajan et al., 2023), bioelectric signals (Cervera et al., 2020; Levin et al., 2017), cell–cell adhesion (Dinet et al., 2021; Schaap, 2021), and local, regional, and systemic regulatory responses (Tagkopoulos et al., 2008).

In this sense, basal cognition is similar to comparative animal cognition studies that attempt to explain interesting observed behaviours in terms of the exhibition of particular cognitive capacities and, subsequently, investigate the possible mechanisms underlying those capacities (Shettleworth, 2010). However, in basal cognition research, both cognitive capacities and underlying mechanisms, in their full variety, are sought after also in non-neural organisms outside of the animal kingdom.

It should be noted that Evo-Devo and basal cognition use the notion of ‘toolkit’ differently. In the former, a developmental toolkit describes shared developmental mechanisms such as the various genes and gene regulatory networks causally involved in various ontogenetic processes that may be common to different taxa (e.g., bilaterians). Such processes are taken to be the mechanistic realisers of developmental phenotypes. Conversely, the basal cognition toolkit comprises the various cognitive capacities that may be common to different taxa, the investigation of which is achieved by searching for shared mechanisms that underpin said cognitive capacities. In both research areas, phylogenetic commonalities and evolutionary conservation are thus explanatorily important. However, their epistemic emphasis differs: in Evo-Devo, the focus is on shared *explanantia* that account for similar or different phenotypes across diverse lineages, while in basal cognition research, the emphasis is on shared *explananda*—specifically, the particular cognitive capacities that mechanistic-comparative research must investigate across diverse creatures, including both neural and non-neural organisms. For basal cognition research, these commonalities that yield adaptive behaviours across taxa, such as shared mechanisms of information processing, storage, and use, are paramount for understanding the evolution of cognition, regardless of nervous system complexity or absence. Rather than merely cataloguing similarities, basal cognition research could use commonalities to explore how cognitive processes evolve and operate across life forms.

At this point, we must stress an important cautionary note that emerges from this parallel: the search for shared developmental toolkits through causal-mechanistic research did not lead to the revolutionary transformation of evolutionary biology that Evo-Devo researchers were forecasting at the discipline’s onset (see Amundson, 2005). Instead, it became part of standard scientific inquiries, now well-entrenched in the horizon of what counts as respectable ‘evolutionary investigations.’ We anticipate that basal cognition research may follow a similar trajectory: after an initial period of scepticism regarding the fruitfulness of searching for putative cognitive capacities and underlying mechanisms across neuronal and non-neuronal species due to theoretical pre-conceptions, we forecast that this endeavour will become a standard procedure in comparative studies of cognition. And, in this sense, contrary to the pronouncements of some basal cognition proponents (e.g., Lyon et al., 2021), comparative mechanistic research alone is unlikely to bring a ‘paradigm shift’ for cognitive science.

One important reason for thinking along these lines is that finding (causal-mechanistic) commonalities in different organisms is only the first step toward asking interesting and nuanced evolutionary questions. In Evo-Devo, the unearthing of *Hox* genes and inquiries into the genetic toolkit, branded by some scholars as a period of “*Hox* mania,” soon led to the “*Hox* Paradox” or the question of how to explain the incredible phenotypic diversity of animals if they all share the same set of developmental genes (Wray, 2001). Why does a coconut crab look so different than a star-nosed mole or a gossamer worm if they share the same basic genetic-developmental toolkit? Renewed explanations of phenotypic diversity, beyond master regulatory genes à la Gehring (1998), became a pressing need in the explanatory agenda of Evo-Devo. If regulatory genes can be co-opted in evolutionary history and acquire new developmental roles, as biologists had to learn after their wide-ranging *explanantia* turned out to be makeshift, then their domains of expression cannot be taken at face value as indicating profound anatomical conservation.^4^ For contemporary Evo-Devoists, it is important to recognize that although developmental regulatory genes are evolutionarily conserved, their interactions might not, and many argue that the evolutionary rewiring of gene regulatory networks has been a pervasive source of morphological change during animal evolution (Kirschner & Gerhart, 2005; Wray, 2001), in addition to other non-genetic, heritable causal processes involved in phenotype production.

Analogously, in basal cognition research, finding common processes and their underlying mechanisms in different organisms, with the goal of understanding their constitution and operation, is only the first step toward asking interesting and nuanced questions about the evolution of such processes and mechanisms. For example, if both communication between individual bacterial cells making up a biofilm and communication between individual neurons in a brain involve electrochemical activity of potassium ion channel gating (Prindle et al., 2015), and such communication in both cases is involved in cognitive capacities such as memory (Yang et al., 2020), then what accounts for the functional variation in the kind of ion channel-mediated memory observed in bacterial biofilms and ion channel-mediated memory associated with neuronal metazoans? In other words, even if mechanisms are conserved, this still leaves us with a central evolutionary question as to why (and how) functional variations in cognitive capacities arose.

This parallel brings us to an important point–a heuristic if you will–that can be mined from the recent history of Evo-Devo and applied to basal cognition research. Although finding taxonomic commonalities in developmental processes or cognitive capacities is of extreme value in furthering our understanding of the evolution of a particular process or capacity, it is just as important to understand differences across taxa given that such differences (i.e., heritable variation) fuel evolutionary processes (e.g., natural selection). Although there has been a tradition of focusing upon organismal differences in both cognitive science and comparative psychology–a spotlight that has unfortunately fed the fire of zoocentric and anthropocentric biases in these fields (Lyon, 2006)–swinging too far in the opposite direction to concentrate primarily on commonalities stands in the way of understanding why the same *general* cognitive process may have evolved differently, taking different forms in different taxa despite being (partially) underwritten by the same mechanisms. We would thus like to emphasise that adopting a fully evolutionary approach involves taking both commonalities and variations of processes and capacities as the proper targets of investigation. The dialectical emphasis on investigating both conservation and diversification has been referred to by Michael Akam as the “Yin and Yang of Evo-Devo” (Akam, 1998), and we think that basal cognition researchers could do very well in adopting something similar as part of their zetetic orientation. Oscillation between investigating commonalities and differences is a core aspect of becoming a fully evolutionary research field–one that avoids unhelpful philosophical polemics and biases and also avoids glossing over the kinds of conservation and variation that developmental and cognitive phylogeny are dependent upon. And, in connection to this point, we arrive at the next parallel.

## The phylogenetic scope of cognition and development: oligoextensionalism versus panextensionalism

When considering the phylogenetic scope of development, one could adopt a priori, definition-first stances regarding their distribution in the tree of life, similar to the prevailing approach for examining cognition. Scholars that choose this path usually assume that both (bundle of) processes are either highly restrictive in their reach, or that they are ubiquitous (i.e., present in *all* living forms). We call these *oligoextentionalist* and *panextentionalist* positions, respectively.^5^ Oligoextensionalist views should be familiar to all of us: viz., cognition exists only in metazoans with proper neural systems, while ‘true’ development is limited to five multicellular clades and only to those clades: animals, plants, red algae, brown algae, and fungi. In contrast, panextensionalist views for either cognition or development are less widespread, but they still can be vigorously found in scientific and philosophical scholarship. Under these lights, *all* living systems are deemed veritably cognitive and with respect to development, *all* living systems are taken to exhibit and embody this process in their life histories, from extremophile bacteria to blue whales.

For oligoextensionalists about development, the purview of developmental biology only covers the growth, differentiation, pattern formation, and remodelling that gives rise to the ‘typical’ adult forms of clonal multicellular organisms (Conway, 2020). In this sense, development can be investigated as a canonical set of events surrounding the changing structures of an organism *en route* towards adulthood or, more specifically, for acquiring the capacity to reproduce. For animals, these events ordinarily comprise fertilization, cleavage, gastrulation, organogenesis (including neurulation), and, for some groups, metamorphosis (Love, 2022). Oligoextensionalism about development is a frequent standpoint for developmental biologists and practitioners of Evo-Devo (e.g., Arthur, 2021).

Contrary to this approach, some Evo-Devoists have called for shedding our preconceptions of what ‘development’ is supposed to refer to and being attentive to the kinds of changes and trajectories that organisms from diverse phylogenetic groups undergo throughout their ontogenetic histories (for an overview, see Minelli, 2021). With this evolutionary diverse window frame, development could hardly be seen as the path from unicellularity to multicellularity, or as a process univocally tied to (sexual) reproduction, or something that ceases upon attainment of an adult-typical form. Indeed, if we look at the diversity of extant organisms and their life cycles, few follow the ordered transformation that turns an egg first into an embryo, then into a juvenile, and finally into an adult (Minelli, 2003, 2021). For panextensionalists, then, development refers to the structured sequence of changes any organism experiences throughout its lifespan, which alter its morphology, physiology, and behaviour. These ontogenetic trajectories can diverge significantly based on the individual or its phylogenetic group—be it a unicellular diatom, the gigantic, single-celled bubble algae, a monocarpic tree, or the so-called ‘immortal jellyfish’ that is able to transdifferentiate back into the sexually immature polyp stage.

For the domain of cognition, being a panextensionalist is one way of subscribing a strong life-mind continuity thesis (i.e., the idea that mind is, and always has been, prefigured in and indissociable from all forms of life). In this sense, some defenders of disparate frameworks such as the theory of autopoiesis, enactivism, and the free energy principle can be counted as panextensionalists regarding cognition (see, e.g., Kirchhoff, 2018; Kirchhoff & Froese, 2017; Maturana & Varela, 1980; Thompson, 2007).^6^ In contrast, oligoextensionalists about cognition differ in which animals, besides vertebrates like mammals and birds, they consider to exhibit cognition (e.g., insects), and they can be quite pluralistic regarding how to construe and assess it (e.g., Bräuer et al., 2020). Nonetheless, they still are to be classified as oligoextensionalists because, across the vast span of evolved lineages constituting the tree of life, they restrict cognition to few clades within the (comparatively speaking) small clade of Metazoa.

Basal cognition is a research program that pushes against oligoextensionalism of cognition, and some of its proponents have explicitly endorsed panextensionalism as an antidote to its biases (e.g., Lyon, 2006). However, we argue that taking a fully evolutionary driven approach means that one cannot subscribe any of the two positions from the get-go, either for development or for cognition. Thinking in evolutionary terms makes us reckon with the fact that we need to situate the evolutionary processes and patterns under study in phylogenetic context. For this, the scope of cognition, in a fully evolutionary view, should be seen as an open question, revisable under the influx of empirical evidence.

In contemporary Evo-Devo, both oligoextensionalism and panextensionalism about development seem to co-exist, but they guide different kinds of empirical investigation. And this is what will help us to bring into focus the upshots and limitations of the second parallel between the study of development and cognition that we have unearthed. Consider for a moment what panextensionalists about development and cognition are actually fighting *against*. Besides resisting ‘adultocentrism’ (sensu Minelli, 2003), panextensionalists about development have challenged a specific a priori tenet that comes with the traditional oligoextensionalist framing, namely that development is something that *only* happens to clonal multicellular organisms, ruling out cases of aggregative multicellularity and the changes that single-celled organisms undergo throughout their life cycles. Likewise, basal cognition researchers impugn a particular tenet of oligoextensionalist framings of cognition, namely that cognitive capacities can exclusively be instantiated by *neuronal* animals. For convenience, let’s refer to the former tenet as ‘strict multicellularism’ and to the latter as ‘strict neuronalism.’

Destabilising these tenets bring some promising investigative avenues. For once, they could give us a more accurate or comprehensive exploration of the bundle of phenomena at stake (i.e., development and cognition). For instance, destabilising strict multicellularism has generated interesting lines of research within Evo-Devo. The idea of development in unicellular eukaryotic organisms may have been completely dismissed not so long ago, but today there is ample evidence for developmental processes in unicellular protists (Bonner, 1967; Gilbert, 2000; Stephenson & Stempen, 1994). Likewise, disavowing strict multicellularism has allowed scientists to investigate bacterial morphogenesis, for example, the mechanisms through which sphere, rod, or spiral shapes are secured (Jiang et al., 2015), or apply the notion of ‘developmental trajectories’ to the formation of biofilms. For instance, Zhang et al. (2021) have investigated the morphogenesis of *Vibrio cholerae* biofilms under confining environments and Futo et al. (2021) have argued that biofilm formation in *Bacillus subtilis* is “a bona fide developmental process” as its shifting genetic expression profiles recapitulate central aspects of its phylogeny.

Even countering strict multicellularism is a promising avenue to try to understand the evolution of multicellularity itself. Arias del Angel et al. (2017) have taken an Evo-Devo perspective on the aggregative multicellularity of myxobacteria. Their rationale is that investigating the diverse evolutionary origins and modes of multicellularity facilitates evolutionary comparative analyses. Such analyses can aid in distinguishing lineage-specific aspects of multicellular evolution (e.g., in animals or plants) from the generic factors and mechanisms underlying the transition to multicellularity across different groups of organisms (Arias del Angel et al., 2017). For scientists with an Evo-Devo mindset, the origin of animal multicellular development, for instance, is sought in the developmental trajectories and developmental potentialities of closely related lineages (Brunet et al., 2019; Dayel et al., 2011; Fairclough et al., 2010; Ruiz-Trillo et al., 2023).

Also, for basal cognition advocates destabilising and resisting strict neuronalism opens up new research avenues. Recent biological research focusing on the behaviour of various unicellular organisms such the ciliate *Stentor coeruleus* (Rajan et al., 2023) and the acellular slime mould *Physarum polycephalum* (Boisseau et al., 2016; Vogel & Dussutour, 2016; for discussion, see also Sims, 2024b) have provided evidence that habituation (a simple form of learning) does not require neuronal processing.^7^ Besides providing concrete reasons for cognitive science and comparative psychology to reconsider traditional implementation constraints that have been imposed upon learning across the board, these empirical discoveries have opened up new and important questions regarding different memory storage mechanisms. Whilst modification of synaptic strength may be one storage mechanism that straightforwardly requires neurons, cellular molecular storage does not (Gershman, 2023). Whether the molecular mechanisms underwriting memory storage in non-neuronal and neuronal organisms are shared is an open question, and–if we are on the right track–one which needs to be addressed along with questions about functional variation in memory and habituation across taxa. That said, resisting strict neuronalism has already hinted that it is worth its weight in salt.

Similarly to the case of multicellularity in Evo-Devo, understanding the evolutionary origins of neurons and nervous systems (e.g., nerve nets) requires investigating non-neuronal organisms within and outside the animal kingdom (Arendt, 2020, 2021). Doing so promises to provide a wealth of information upon which to make empirically based inferences about the economisation of organismal organisation that early nerve nets provided for basal metazoans (Keijzer et al., 2013). Likewise, recent work has found neuropeptide homologs in choanoflagellates, thus pointing to the pre-metazoan onset of their signalling functions (Yañez-Guerra et al., 2022), a relevant piece of information for understanding how they later were co-opted in nervous system evolution.

In general, evolutionary-driven approaches are promising for transcending biases and steering clear of reifying extreme positions (i.e., both oligoextensionalism and panextensionalism). Uncritically subscribing to panextensionalism, the assumption that development or cognition pervades all organisms, could impede scientific hypothesis construction and testing and, more generally, obscure the intricate relationships between the processes and capacities subsumed under these broad umbrella terms. In particular, if cognition is stipulated as a feature of all organisms, past and present, the largest evolutionary puzzle then resides in the origin of life itself (which would also be identical to the root of cognition), rendering subsequent inquiries on the evolution of cognition as mere complexifying elaborations on a common theme.

With this we arrive to another important cautionary note. In both developmental and cognitive sciences, oligoextensionalism and panexensionalism represent opposing extreme positions: development and cognition occur only in some forms of life (clonal multicellular and neuronal respectively) *vs*. development and cognition occur in all forms of life. Although extreme positions can often ignite and arouse interest in a particular field of research—especially for newcomers to a field or in the early days of a research field—radical positions often turn on deploying definition-based approaches and, as such, disputes tend to take the form of recalcitrant exercises in redescription—enter the many-headed hydra. However, as empirical discoveries are made, extreme positions can sometimes give way to what might look like a ‘golden mean.’ As Stephen J. Gould writes of the eventual closing of the gap between eighteenth century preformationists and epigeneticists schools of thought regarding heredity and embryonic development:

Modern genetics is about as midway as it could be between the extreme formulations of the eighteenth century. The preformationists were right in asserting that some persistence is the only refuge from mysticism. But they were mistaken in postulating preformed structure, for we have discovered coded instructions. […] The epigeneticists, on the other hand, were correct in insisting that the visual appearance of development is no mere illusion. (Gould, 1977, p. 18)

Our ﻿point is not to suggest that something like a golden mean should be the aim for oligoextensionalism or panextensionalism with respect to cognition and/or development^8^; rather, we would like to argue that deciding between these extremes is a matter of carrying out observations, formulating testable hypotheses, performing experiments (behavioural, structural, and molecular analysis), and letting the results of such experiments help scientists determine which organisms engage in which forms of cognitively driven behaviour and/or developmental processes (if at all) *and* how those specific forms might share commonalities or differ from those exhibited by other taxa. It was a similar kind of slow yet, as we now recognise, fruitful empirical process that led to the golden mean between preformationism and epigenesis which contemporary genetic research represents. This kind of process is core to a fully evolutionary approach. Similarly, we may expect that the polarising tension between oligoextensionalism and panextensionalism with respect to cognition will likely be mediated by taking a dialectical evolutionary-driven approach. Such an approach views working definitions as starting points that can be adjusted as needed with new empirical discoveries, rather than as fixed endpoints requiring theoretical defence.^9^ Philosophical adherence to a priori, fixed conceptions of cognition, in both oligoextensionalist and panextensionalist camps, can hinder the formulation of hypotheses that diverge from these conceptions or obscure evidence that contradicts them. Basal cognition researchers would do well to remember this.

In fact, as philosophers of Evo-Devo have judiciously argued, it is often more productive to move beyond offering univocal definitions of scientific concepts and instead characterise the explanatory agenda associated with them. This involves identifying the specific questions being addressed by the relevant epistemic communities, delineating the research horizon of salient problems they are confronting, and clarifying the success criteria for their resolution. For instance, Brigandt and Love (2012) explored this approach in their discussion of ‘evolutionary novelty,’ and Nuño de la Rosa (2017) applied similar reasoning to the concept of ‘evolvability.’ The key insight from these analyses is that conceptual diversity can actually stimulate scientific research. Accordingly, basal cognition research might benefit from embracing different theoretical perspectives on what ‘cognition’ is that could foster interdisciplinary collaborations in addressing its core agenda: understanding the evolution of specific cognitive capacities (e.g., memory, learning, decision-making, and anticipation) and their mechanistic underpinnings across the tree of life.

In the next two sections, we delve deeper into the promising avenues and cautionary notes that sound phylogenetic thinking, another stepping stone *en route* to a fully evolutionary orientation, could bring to future research on basal cognition.^10^

## Thinking phylogenetically: homologies, homoplasies and convergent evolution

In fields thoroughly grounded in evolutionary considerations, like Evo-Devo, multiple ‘styles of thinking’ are employed to study evolution. These include, in addition to the causal-mechanistic pursuits discussed in Sect. 2, the well-established ‘population thinking,’ which focuses on uncovering the evolutionary forces driving changes in trait frequencies across generations, as well as ‘tree thinking,’ essential for launching comparisons across species and mapping evolutionary relations, and ‘homology thinking,’ crucial for comprehending how organismal traits are individuated during development and how they can exhibit quasi-independence in their evolutionary trajectories (Wagner, 2016). Explaining the wide range of characters across the tree of life, including ‘cognitive characters’ (for discussion, see Figdor, 2022, 2024a), requires understanding various processes that have given rise to diversification of extant life and properly contextualizing them within their phylogenetic history. And for this, distinguishing between *homologies* and *homoplasies* is of paramount importance.^11^

By comparing lineages and organisms from different species, evolutionary biologists bring together tree thinking and homology thinking and aim to uncover ancestral relationships that underlie the shared identities of certain characters (i.e., homologies), and this is useful, say, in tipping the balance when gauging competing models of trait evolution in particular phylogenetic sequences. However, also cases of convergent evolution are epistemically significant in these comparisons, as they strengthen and help confirm hypotheses about adaptation (Currie, 2013), to give an example. Finding similar phenotypes in different organisms may be explained by retention from common ancestry (homology), but a careful phylogenetic appraisal may instead reveal that they are independently derived, due to convergence or parallel evolution, or that they experienced reversal to a plesiomorphic state. Such examples of homoplasy present opportunities for Evo-Devoists to discover potential underlying developmental mechanisms and components that may have been redeployed to produce the prima facie ‘same’ phenotypes (Wake et al., 2011). In this vein, Evo-Devoists have come to the realization that the properties, dynamics, and organizational patterns of development make certain analogous structures more likely to evolve (Minelli, 2019).

Drawing inspiration from Evo-Devo, a set of promising avenues for the field of basal cognition could be carved out by embracing ‘tree thinking’ and ‘homology thinking’ and benefiting from the noetic and explanatory resources they afford for their evolutionary investigations (Hall, 2012; Wagner, 2016). Along these lines, basal cognition research would benefit from differentiating between homologies and homoplasies when investigating cognitive traits and cognitive capacities. Discerning independent evolution and marking it off from bona fide uninterrupted (homologous) continuities, which cannot be presumed without the right kind of phylogenetic evidence, is epistemically decisive for avoiding ill guided inferences and unassured extrapolations.

The panextensionalist framing of basal cognition research that we explored in the preceding section unwittingly assumes that the similarities in the cognitive toolkit (see Sect. 2) are indicative of *homology*. For if the same cognitive capacities manifest in all extant life forms, from cyanobacteria and thermodesulfobacteriota bacteria to telonemids, spiny gulfweed and zombie fungi to hominids, it implies their presence in their most recent common ancestor—in this case, the putative LUCA, or ‘Last Universal Common Ancestor.’ However, merely uncovering similarity is insufficient to substantiate a particular evolutionary hypothesis concerning homology or homoplasy, for both insinuate similarities that require elucidation in the first place. Proper phylogenetic contextualization is a non-negotiable provision for putting these kinds of evolutionary inquiries on a productive track. This underscores a cautionary caveat: homology and homoplasy demand thorough and explicit assessment in basal cognition research, not mere surmises drawn from observing similar behaviours in organisms whose evolutionary kinships remain unclear (Figdor, 2024a, 2024b). With this, we do not want to rule out homology in (molecular, behavioural, etc.) traits that are related to the so-called basal toolkit of cognitive capacities, but rather point to the need to solidify the evidential apparatus for making such sweeping claims.

In extant organisms, we could witness similar capacities like memory, decision-making and anticipation, but it is entirely possible that some could be the product of common ancestry (e.g., conserved through uninterrupted negative selection towards variants that diminish their sizable contributions to organismal fitness) while others could have independently evolved in the different lineages giving rise to them. This is something that basal cognition advocates should evaluate and take up in their inquests to counter strict neuronalism. In this sense, probing the evolution of homoplasies can also be valuable for the basal cognition programme. For instance, multiple cases of convergent evolution have been uncovered in vertebrate nervous systems (for overviews, see Nishikawa, 2002; Strausfeld & Hirth, 2016) and some Evo-Devoists have adduced evidential support for the hypothesis that bilaterian central nervous systems evolved independently in chordates and protostomes (Holland et al., 2013; Martín-Durán et al., 2018). Correspondingly, convergent evolution needs to be investigated and cannot be ruled out for putative cognitive traits and even larger cognitive capacities in non-neural organisms from disparate lineages. For if, as basal cognition supporters claim, echoing Shettleworth's (2013) definition, cognition “is comprised of sensory and other information-processing mechanisms an organism has for becoming familiar with, valuing, and interacting productively with features of its environment in order to meet existential needs” (Lyon, 2020, p. 416), why couldn’t processes involved in acquiring, storing, and using information from diverse environments have evolved more than once in the history of life?

Moreover, there is no demerit whatsoever in a trait or process being the product of independent evolution. For example, fossil and molecular evidence suggest that megaphyllous leaves in euphyllophytes (ferns, gymnosperms and angiosperms) and microphyllous leaves in lycophytes (clubmosses, spikemosses and quillworts) may be homoplastic, having emerged several times in plant evolution (see, e.g., Corvez et al., 2012; Harrison et al., 2005). But this does not mean that these remarkable organs/structures linked to photosynthesis and transpiration found across euphyllophytes and lycophytes are not *leaves* or that we shouldn’t call them ‘leaves.’ Why cognition could only be called ‘cognition’ if it is tied to the lineages traditionally studied by cognitive science, as oligoextensionalists would have it? Or, as panextensionalists presume, if it is batch of capacities that all and every single organism instantiates? What are the arguments and the evidential basis to not even entertain the possibility of convergent evolution of cognitive traits and capacities throughout the tree of life?

Importantly for our foregoing comparisons, even when granting that (clonal multicellular) development evolved convergently in, say, plants and animals, we don’t have a problem to still describe their bundles of processes related to events like morphogenesis and differentiation as ‘development.’ Why should things be so different, borderline sui generis, when deliberating about cognition? We argue that not even countenancing, even if remotely, the possibility that certain cognitive processes and capacities might not be the outcome of an evolutionary singularity is antithetical to biological thinking. We don’t assert that plant development is not development because it is a product of independent evolution from animal development. Authors like Meyerowitz (2002) have argued that it is precisely because they are convergent that we can gain a lot of insight into what is truly general about development by comparing them (and at the same time, these comparisons accentuate the fact that certain processes are genuinely unique in both groups and cannot be generalized).^12^ This is another lesson, at the same time exciting and daunting, that is uncovered by our analysis.

In light of this backdrop, it would be advantageous for scientists and philosophers interested in basal cognition to give greater consideration to convergent evolution and homoplasies more generally in their research and especially in their emerging theoretical frameworks. For instance, in the recent double special issue in *Philisophical Transactions of the Royal Society B* on basal cognition, only one article has a section devoted to convergent evolution (Moroz et al., 2021), but it is restricted to treating neural systems in animals, and two other articles only cursorily mention it (Baluška & Mancuso, 2021; Boussard et al., 2021; but see Baluška & Mancuso, 2009). Likewise, none of the articles allude to homoplasy.^13^ This infrequent treatment, we believe, provides some evidence that this notion is not taken to be as important as homology—something that we think might be the by-product of their strong assumption of Darwinian continuity (Levin et al., 2021). Our suggestion for basal cognition research is not that it should simply be aware of homoplasies when countering strict neuronalism, but rather that it should countenance the importance of this evolutionary route and the frequency with which it happens and complement a more linear view of cognitive evolution with scenarios of parallel and convergent evolution of cognitive traits and capacities. A nice example in this direction is the recent discussion by Romanova and Moroz (2024) on convergent evolution of gravity sensors in unicellular protists and animals (e.g., Müller vesicles and statocysts), and the additional parallel evolution of gravisensory systems in basal metazoans. In the motley frame painted by the authors, gravitational sensitivity and the ensuing locomotory integrative systems cannot be understood through a view of linear cognitive evolution. With this in mind, the evolution of cognitive traits and capacities, if treated with suitable phylogenetic tools and context, cannot be construed as a linear march of progress, as we will showcase with the next parallel.

## Complexification, simplification and the loss and gain of traits

By embracing phylogenetically-oriented thinking, besides being mindful of convergent and parallel evolution and taking appropriate steps for distinguishing between homologies and homoplasies across different (far-away and close) branches of the tree of life (see also Figdor, 2023, 2024b), the research undertaken under the basal cognition umbrella could profit from investigating *phyletic evolution* more closely (i.e., evolution within particular lineages). In this sense, it is important to stress that losses and gains of traits can come about, and the evolution of cognition should not be an exception on this regard.

As a general remark, over the course of phyletic evolution, one could attest relative complexification or simplification in the clades under study. When confronted with changing macroevolutionary patterns of biodiversity, biologists often appeal to “major transitions in evolution” (Szathmáry & Smith, 1995) and more strictly to “major transitions in individuality” (Michod, 1999) in an attempt to explain them. Roughly, such transitions identify hierarchical shifts in complexity that involve the emergence of higher-level units with novel (unit-dependent) features that arise from the coming together of lower-level entities. Such transitions have been interpreted to suggest that, in some lineages, there is an evolutionary trend for organisms to become more complex vertically (i.e., increasingly nested) and horizontally (i.e., possessing a larger number of different component parts). Primary examples are transitions from prokaryotic to eukaryotic life and from unicellular to clonal multicellular eukaryotes. In this regard, Evo-Devoists ask, for instance, if there is co-evolution of certain organelles (e.g., Woronin bodies of the *Ascomycota phyla*) and multicellular complexity (Jedd, 2011; for discussion on complex metazoan multicellularity, see Nejad Kourki, 2022).^14^

Although organismal complexification is one way in which evolution proceeds to produce variation in particular lineages (Bonner, 1998; McShea, 2016), it is not the only way. The role of simplification is becoming increasingly recognised as a major driver of trait diversity within Evo-Devo and evolutionary biology more generally (e.g., O’Malley et al., 2016; Schoch, 2014). Secondary simplification, as the term suggests, refers to the idea that the evolution of traits as observed in some extant species may have involved a trajectory from complex to simple *or* from more complex to less complex traits. Such simplification may involve genetic streamlining (i.e., deletion of genes or gene complexes) and/or the gradual loss of anatomical structure or behavioural phenotypes (O’Malley et al., 2016). One example of interest for Evo-Devoists of how secondary simplification has played a role in evolutionary diversification can be found when looking at the phylogenetic relationship between choanoflagellates, filastereans, and metazoans.

Choanoflagellates are currently recognised as the closest living relative of metazoans and this makes them of particular interest to biologists investigating the transition from unicellularity to multicellularity (King et al., 2008; Ruiz-Trillo et al., 2023), as we mentioned in Sect. 3. One of the various routes to multicellularity that has been hypothesised (there are at least 11 for each of the major eukaryotic clades), suggests that animals originated from a colonial stage reminiscent of present-day choanoflagellates (for other alternatives, see Ruiz-Trillo et al., 2023).^15^ Genomic comparison between choanoflagellate *Monosiga brevicollis* and various animals has been used to infer that their last common ancestor shared some cell adhesion and cell–cell communication gene complexes but failed to possess many other significant signalling pathways and transcription factors found in animals and important to their multicellular development (O’Malley et al., 2016). However, recent genomic sequencing of *Capsaspora owczarzaki*, a unicellular opisthokont belonging to Filasterea—a close sister clade to both choanoflagellates and animals, has revealed that they share developmentally important cell adhesion genes complexes (i.e., integrins) and transcription factors with animals which were lost in choanoflagellates (Sebé-Pedrós et al., 2010). This has led biologists to suggest that a secondary simplification may have occurred. It is likely that the last common ancestor of filastereans, metazoans, and choanoflagellates possessed integrins and various transcription factors that were lost in choanoflagellates but retained and repurposed in metazoans.

Many more examples of Evo-Devo research and discussions on secondary simplifications could be cited (e.g., Hannibal & Patel, 2013; Hoekstra et al., 2018; Virágh et al., 2021), including cases related to nervous system evolution in metazoans (Roth et al., 1997; Tosches, 2017). The implications of secondary simplifications are an underexplored topic in basal cognition research, a programme which, as we saw in the previous section, has been grounded on an assumption of gradualism and complexification over evolutionary timescales; there is a tendency to think in linear terms.

One additional moral of the story, to paraphrase Clark (1999), is that ‘simple’ does not always mean evolutionarily primitive (see also Godfrey-Smith, 2017). In fact, as some Evo-Devoists have argued, *basal* taxa are not necessarily the best available stand-ins for ‘ancestors’ (Jenner, 2006), and these, due to being adjacent to the root or in proximity to it in a (rooted) phylogenetic tree, are often incorrectly interpreted as having undergone less change and thought to be more “primitive” or “ancestral” than subsequently branching lineages (Crisp & Cook, 2005; Gregory, 2008). It may be beneficial for basal cognition researchers to ponder this when selecting their model and study organisms.

Flipping the coin and chewing over complexification from the viewpoint of major transitions in individuality that we sketched above, as these also need to be weighed in, what might the processes of vertical complexification mean for basal cognition? In any given instance, should we shift the locus of analysis to the higher biological level under the assumption that it becomes the more ‘dominant’ level of selection that constrains the behaviour of the lower levels, or should we continue to focus on lower levels despite their being less autonomous? In other words, major transitions in individuality are conceptually and empirically distinct from major transitions in cognition and although the later have been explored in detail (Barron et al., 2023; Ginsburg & Jablonka, 2019, 2021), the implications of the former have gone under the radar to the detriment of basal cognition.

Complexification and simplification are just two evolutionary scenarios that involve gains and losses of traits in evolutionary history. However, the parallel that we are delineating for basal cognition research reaches beyond these scenarios. We want to underscore the overall importance of gains and losses of traits in evolutionary investigations, something that deeply affects the types of inferences and explanations that could (or shouldn’t) be drawn from the species chosen for scientific study. In this domain, Evo-Devo has many lessons to bestow to basal cognition research. For one, Evo-Devoists argue that both losses and gains are important for explaining evolution (e.g., Cannell et al., 2020).^16^ Evolution is thus sometimes described, from a phylogenetic perspective, as a “never-ending cycle of gains and losses” (Keller & Delaux, 2022). In this tenor and building from the *corpus* of knowledge on the evolution of other biological features, we forecast that both gains and losses will prove to be salient when addressing the evolution of certain cognitive traits present in extant organisms (and perhaps for the evolution of some of the cognitive capacities in the more encompassing basal toolkit).

Furthermore, there are a few extra things to bear in mind. Evo-Devoists have argued that we should fight our unintentional bias toward interpretations that favour trait gains over losses (Church & Extavour, 2020) and have shown than the loss of traits is not an all-or-nothing phenomenon, but rather traits are lost to varying degrees along a so-called “trait loss continuum” (Sadier et al., 2022). Likewise, multiple gains and losses of characters in related taxa are not at all uncommon, something that could be partly accounted for through the Evo-Devo notion of ‘deep homology,’ “pointing to the persistence of developmental potentials that are not always expressed” (DiFrisco, 2019). The loss and gain of cognitive characters could also be subject to evolvable potentials that are not always expressed, but that could easily tilt in one or the other direction in related taxa. In sum, the loss and gain of traits (including complexification and simplifications) are intricate *explananda* and simultaneously powerful *explanantia* in fully evolutionary fields. Recognising their significance and giving them adequate treatment would help ensure that basal cognition research develops in a manner that is consistent with being a fully evolutionary approach.

The final remark that we would like to advance on this theme is that countenancing losses and gains in cognitive evolution forces us to reckon with the fact that in some lineages, and only in those lineages, there might be cases of unique traits (i.e., autapomorphies). In this sense, a fully evolutionary research program should not avoid questions about cognitive uniqueness, for instance, in *Homo sapiens*, but also in other non-human organisms, including non-neuronal ones. As an illustration, if enough evolutionary evidence allows us to call (certain) fungi ‘cognitive,’ they might be unique in different ways that, for instance, birds or humans might fail to be. Regarding this issue, we think that basal cognition researchers could follow Shettleworth (2010), who claims that in the evolutionary study of cognition

[…] neither blanket anthropomorphism nor complete anthropodenial is the answer […]. When it comes to comparing human cognition with that of other species, it is most likely that—just as with our genes and other physical characters—we will find some processes shared with many other species, some with only a few, and some that are uniquely human. (p. 19)

As we mentioned in Sect. 2, the (Evo-Devo-inspired) Yin and Yang of investigating conservation and difference in evolution, including gains and losses of traits that make certain lineages stand out, should be central for basal cognition research.

## Concluding remarks

We began this paper by distinguishing two distinct manners of investigating the scope of biological cognition. The first of which, the definition-first approach, engages in the philosophical practice of constructing and amending definitions of cognition and it is primarily guided by a priori intuitions of what cognition is and where it is found in the biological world. Using an evolutionary-driven approach represents a second manner of investigating cognitive scope. While working definitions are a starting point, this kind of approach leads with what empirical investigations and phylogenetic contextualization reveal about the presence or absence of various cognitive capacities in the organisms investigated. Here the question of cognitive scope is not a question that reduces to one of strictly characterising and meeting a definitive ‘mark of the cognitive’ but is rather grounded in a question about how certain cognitive capacities that we recognise as such have evolved. The programme of basal cognition embodies this latter kind of approach and has the potential to avoid some of the stalemates and unending semantic battles–our familiar many-headed hydra–that have plagued definition-first approaches. In this paper, we have uncovered four parallels between the fields of Evo-Devo and basal cognition: (i) the search for shared, conserved toolkits and the importance of comparative causal-mechanistic research; (ii) panextensionalist framings to counteract the evolutionary limitations of oligoextensionalism about development and cognition; (iii) the implementation of phylogenetic thinking to uncover homologies and homoplasies (especially possible convergent routes of evolution); and (iv) the possibility to study losses, gains and uniqueness of traits, including complexification and simplification in particular lineages.

In its onset, Evo-Devo, like basal cognition today, was embroiled in controversy due to its departure from received views of development and its evolution. Evo-Devo eventually addressed many *explananda* regarding the (somewhat shared) causal-mechanistic underpinnings of morphogenesis and how development matters for accounting for evolutionary dynamics. In giving treatment to each of these four parallels, we have aimed to show that much of the current scepticism towards basal cognition and the idea of fruitfully investigating of cognition outside of the animal kingdom may also be unwarranted, or at least it should be tempered down. From each of the parallels identified, related cautionary notes for basal cognition have been drawn out (e.g., the need to complement investigation on evolutionary conservation with research on evolutionary divergence and differences, the pitfalls of trying to adjudicate between the antipodes of panextensionalism and oligoextensionalism about cognition in an empirically detached manner, the lack of attention paid to convergent evolution and the loss and gains of traits, notably secondary simplification, and assuming that ‘basal’ means ‘primitive’). These are cautionary notes that we hope will provide some guidance for this exciting new research programme on its way to becoming a fully evolutionary field.

Let us return to the examples of scepticism. As we saw, one form argues that if there is no strong evidence of similarity between what is being called “learning” or “memory” in non-neuronal organisms is and what cognitive scientists are interested in, there is no reason to deem those processes in non-neuronal organisms as such. To this, we respond: Firstly, there is no unified consensus within cognitive science regarding what cognition is, and the interests of cognitive scientists vary significantly (Cf. Sims, 2021). Secondly, even if there was consensus, using the interests of cognitive scientists as an exclusive metric is problematic given that the historical focus of this field has been on humans and non-human animals. Insisting that all instances of memory, learning, and decision making across taxa must resemble those of humans or of non-human animals is to be under the spell of oligoextensionalism. Moreover, just how similar a process must be to be considered *similar enough* to what cognitive scientists are interested in is also a vague matter. For instance, if *P. polycephalum* (slime mould) exhibits habituation in experimental conditions that rule out sensory fatigue (which it does), and habituation is widely acknowledged as a form of learning in animals, how much more similar must its habituation be to that in the rodents, corvids, or primates to count as relevant to the interests of cognitive science? As empirical research in basal cognition advances, some of this scepticism is likely to increasingly resemble the mid-20th-century resistance among developmental and evolutionary biologists to acknowledging developmental similarities across diverse taxa—a stance now seen as an artifact of pre-theoretical bias.

The second form of scepticism argues that even when basal cognition researchers successfully use feedback (cybernetic) models to represent adaptive and flexible behaviour in non-neuronal organisms, these models omit the phylogenetic and conceptual connections between such behaviours and those of organisms that are already recognised as cognitive. Our response is this: cybernetic models, like all scientific models (e.g., systems biology models), are abstract and idealized simplifications (for discussion, see, e.g., Andrews, 2021). By abstracting away from the complexity of target phenomena, models can serve as proof-of-principle tools for capturing cognitive principles in a, say, mathematically or empirically tractable manner. They do not provide evidence for cognition in a specific target system but buttress our understanding of possible shared mechanisms underlying observed behaviour. Thus, while Figdor (2024b) is correct that current basal cognition’s models usually omit phylogenetic and conceptual relationships, this omission is not a flaw on the part the basal cognition research programme or their cybernetic models, but rather a limitation inherent to the specific purpose of such models. The difficult task of elucidating such relationships instead falls within the domain of bioinformatics, comparative genomics, molecular phylogenetics and the models they deploy for cross-species comparison and phylogenetic reconstruction. A fully evolutionary approach, like the one basal cognition research could foster, would be able to complement comparative-mechanistic research with sound phylogenetic reconstructions and evolutionary-contextualized inferences. So basal cognition researchers could also challenge this line of scepticism in the future.

Importantly, since behaviour (cognitive or otherwise) cannot be reduced to gene activity, identifying phylogenetic and conceptual connections between behaviours of different taxa also requires taking reciprocal organism-environment dynamics into account (Baedke et al., 2021; Sims, 2024a). Considerations from Evo-Devo thus have a further significant role to play here in tandem with basal cognition research.

Finally, scepticism based on definitions of cognition, takes for granted that there is one agreed upon meaning (i.e., a natural kind) and views the job of empirical research of one of finding instances that satisfy this definition. Not only is there no such consensus, but, as our analysis in this paper has highlighted, putting all of one’s eggs in a rigid definitional basket might hinder and distract from hypothesis-driven investigation. As we have shown, investigating conserved toolkits, offsetting strict multicellularism, implementing phylogenetic thinking, and studying losses, gains and unique traits have yielded valuable insights in Evo-Devo. Similarly, an evolutionary approach to cognition can benefit from moving beyond using a priori intuition as an ends that prematurely constrain enquiry rather than as a means to further discovery. Although some degree of scepticism can foster scientific rigor and should always be weighed to evaluate scientific research programmes, scepticism based solely on (contested) definitions rarely contributes meaningfully to science in an empirically profitable manner.

Placing the scope question aside, we would like to end briefly with a few additional questions for future research that we believe may also be useful for basal cognition or any other approaches to cognition in which evolution is central.

- How might **exaptation** (i.e., the co-option of an adaptive trait to play a different function than its original one) have played a role in the evolution of some cognitive capacities or cognitive mechanisms in particular linages that we see today and how can this be studied?
- How can the practice of **good taxon sampling** (i.e., collecting data across major branches of the tree of life for comparisons at different degrees of taxonomic distance) be implemented in basal cognition to both avoid the tendency to overgeneralise and support specific generalisation across phylogenetic branches when evidenced? Better phylogenetic resolution has changed our understanding of neural evolution in animals (Hejnol & Lowe, 2015; Martín-Durán & Hejnol, 2021), and this is a necessary step for the advancement of basal cognition research as well.
- How can we build **robust frameworks for launching meaningful comparisons** across (neuronal and non-neuronal) species that (might) exhibit cognition? A good pointer in this direction might be how *reproduction*, a widespread but radically divergent biological process across phylogeny, is studied comparatively (see Fusco & Minelli, 2019).
- Can the kind of **mesocosm experiments** deployed in ecology be used in basal cognition research to investigate context-sensitive, cognitively-driven behaviours that might be otherwise supressed in largely artificial lab settings?
- How can scientists study how **cognition could affect patterns and dynamics of phenotypic evolution** (similar to how development is taken to be important in Evo-Devo), complementing the studies on the evolution of specific cognitive repertoires and capacities?

Although much work still remains to be done, basal cognition shows great promise for developing into a fully-fledged field of evolutionary research.

## Footnotes

1 It should be emphasised that basal cognition is neither a type of cognition (e.g., “minimal cognition”), nor should this specific research programme be confused with or overgeneralised to include any and all research that has previously investigated the evolution of cognition in invertebrates or other structurally simple neuronal metazoans.

2 Inspired by the work of comparative psychologist Sara Shettleworth (2010), basal cognition researchers offer a “provisional working definition” of cognition as “the sensory and other information-processing mechanisms an organism has for becoming familiar with, valuing, and interacting productively with features of its environment […] in order to meet existential needs, the most basic of which are survival/persistence, growth/thriving, and reproduction” (Lyon et al., 2021, p. 4). Importantly, these authors take this operational characterization to be a “phyletically neutral definition” (p. 4) that serves merely as a heuristic framing to drive research forward without prescribing from the armchair which organisms should or should not be empirically tested as possessors of cognitive capacities. As provisional working definitions are not intended to determine or fully settle the question surrounding the scope of cognition, we believe that basal cognition research diverges from the definition-first approach and aligns well with the *ethos* underlying the evolutionary-driven approach.

3 To complicate things further, some scientists have argued that developmental processes like morphogenesis, tissue patterning and regeneration can be productively studied using the tools and concepts from cognitive and computer science (e.g., Manicka & Levin, 2019; Watson & Levin, 2023). Moreover, some scholars maintain that all of development boils down to being cognitive processing. Although we believe that cognition and development can be disentangled, we won’t pronounce ourselves on the issue of development = cognition in this article. What is important for our purposes here is only to highlight how the evolution of what is taken to be ‘development’ by Evo-Devoists has been approached in their evolutionary investigations and how this could inform research in basal cognition.

4 Returning to our example, it must be said that *Hox* genes have been shown to be much more complex than originally assumed, and their similarities (e.g., in terms of synteny) have been overemphasized (see, e.g., Duboule, 2007). In a similar vein, the extent to which a shared genetic toolkit (made up of individual genetic ‘tools’ with particular functions) has been conserved across the animal kingdom perhaps has been overstated. This was, however, a useful idealization for propelling the field of Evo-Devo forward.

5 The prefix “oligo” comes from the Greek word ὀλίγος, signifying “few” or “little,” and the prefix “pan” stems from the Greek word πᾶν, meaning “all” or “every.”.

6 Being a panextensionalist about cognition is not the same as endorsing ‘biopsychism.’ This position advocates that all life is not only cognitive but also *sentient*, capable of *feeling* and not merely responsive to (affective valences-devoid) sensory stimuli in experiencing the world (Thompson, 2022). Likewise, panextensionalism is perfectly distinguishable from ‘panpsychism,’ a vantage point that takes all matter (life and non-life alike) to be minded. Panextensionalism about cognition only posits that all forms of life engage, in some form or another, in cognitive processes (without this necessarily implying sentience or autonoetic consciousness).

7 Habituation is characterised by a progressive decrease in behavioural response to a stimulus as a result of repeated encounters with the same stimulus.

8 To be sure, Gould did not advocate for the position that a golden mean should be sought for ending polar theoretical positions. Our point is just to illustrate that a middle ground is one of the possibilities that can emerge from continued empirical investigation. In the case of cognition, what might a golden mean look like? Perhaps something like ‘polyextensionalism,’ the idea that neither few animals nor all organisms engage in behaviour that is driven by cognition. Polyextensionalism, whose prefix is derived from the Greek πολύς signifying “many” or “much,” implies that even though many organisms might qualify as exhibiting cognition, working out which of them actually do cannot be settled in an a priori fashion. It is perhaps the most challenging position, detached from the comfort of restricting cognition to only a few animals or readily assuming its totalizing distribution in all life, but it is probably the position closest to the scientific *ethos*: we cannot know in advance which organisms have cognition unless we set out to study them empirically under the right conditions.

9 Along similar lines, Keijzer (2021) defends a world-to-concept direction of fit for grasping cognition instead of a concept-to-world direction of fit that heavily relies on intuitions.

10 We must make explicit here that discussions about homology, homoplasy and the loss and gain of traits, which will be broached in the coming sections, are not proprietary to Evo-Devo, but are part of the epistemic toolkit of ‘comparative biology’ at large. In this sense, some authors working in the tradition of comparative cognition—but not in the basal cognition research programme —have already mobilized some of these insights to explore the evolution of cognition within the animal kingdom (see, e.g., Halina, 2023; Schnell et al., 2021).

11 Homology entails an identity relationship between traits in different species based on historical continuity, irrespective of realized form and function (e.g., human arms and bat wings are two characters states of the same homologous character, namely the tetrapod forelimb). In contrast, homoplasy refers to the relationship between traits in two or more unrelated species that bear similarity that is not explainable by recourse to common ancestry. Homoplasy can occur by convergent evolution (e.g., when similar selective pressures are at play and yield *analogous* traits), by parallelism (e.g., when shared developmental mechanisms independently produce comparable traits), or by reversals and atavisms.

12 There is indeed untapped epistemic potential for basal cognition research with regard to convergent evolution. For instance, Powell and Mariscal (2015) contend that, given the appropriate circumstances, instances of convergent evolution can serve as legitimate *natural experiments*, allowing us to draw well-founded inferences about the counterfactual stability of certain evolutionary outcomes. In this sense, by uncovering convergent evolution, scientists investigating basal cognition could peer into what is constrained or more likely to happen in the evolution of organisms that exhibit cognition in several replicate lineages.

13 In a more recent basal cognition special issue, convergent evolution of the chemical connectome is explored by Moroz and Romanova (2023). This is a move in the right direction.

14 In contrast, to give an example, evolutionary biologists also ponder on the causal factors that explain why aggregative multicellular organisms have “stayed simple” (see Márquez-Zacarías et al., 2021).

15 In relation to the parallel that was exhumed in Sect. 3, Ruiz-Trillo et al. (2023) contend that unravelling the evolutionary origins of animal multicellular development necessitates a systematic effort to sample and probe hitherto unexplored *unicellular lineages* closely related to the animal kingdom.

16 The issue of gains and losses of traits brings to the table questions concerning the irreversibility of certain evolutionary trajectories, something that is deemed to be one of the biggest unsolved challenges in the explanatory agenda of Evo-Devo (see Minelli, 2015).
